# Psychosocial stressors prior to Down Syndrome Regression Disorder: findings from two referral clinics

**DOI:** 10.3389/fpsyt.2026.1799799

**Published:** 2026-07-07

**Authors:** María del Carmen Ortega, Felipe Ortuño, Diego Real de Asúa

**Affiliations:** 1 Department of Psychiatry and Clinical Psychology, Clínica Universidad de Navarra, Madrid, Spain; 2 Department of Psychiatry and Clinical Psychology, Clínica Universidad de Navarra, Pamplona, Spain; 3 Health Research Institute, Navarra (IdiSNA), Pamplona, Spain; 4 Department of Internal Medicine, Adult Down Syndrome Outpatient Clinic, Hospital Universitario de La Princesa, Madrid, Spain; 5 Biomedical Research Foundation of the Hospital Universitario de La Princesa, Instituto de Investigación Sanitaria Princesa, Madrid, Spain

**Keywords:** Down Syndrome Regression Disorder, psychosocial stressors, adverse life events, regression, down syndrome

## Abstract

**Objective:**

To examine the prevalence, categories, and timing of psychosocial stressors preceding Down Syndrome Regression Disorder (DSRD) onset, and to characterize the clinical presentation in a cohort of adolescents and adults evaluated at two specialized referral clinics.

**Methods:**

We conducted a retrospective cohort study including all individuals diagnosed with DSRD between 2021 and 2024. Psychosocial stressors occurring ≤ 6 months before symptom onset were identified using a standardized coding matrix and independently rated by two blinded coders. Sociodemographic, clinical, and functional severity variables were summarized using descriptive statistics and exact 95% confidence intervals.

**Results:**

At least one adverse psychosocial stressor was identified in 91% of the cohort (31/34; 95% CI 76–98). The most frequent categories were bereavement or illness of a close person (35%; 95% CI 20–54) and separation or displacement of a significant figure (32%; 95% CI 17–51). Social withdrawal (100%; 95% CI 90-100), psychomotor slowing (97%; 95% CI 85-100), and loss of previously acquired abilities (94%; 95% CI 80-99) were the most common symptoms at initial assessment.

## Introduction

Down Syndrome Regression Disorder (DSRD) is characterized by the subacute or acute loss of previously acquired skills in individuals with Down syndrome (DS), accompanied by emotional, behavioral, and motor changes. It typically emerges during adolescence or adulthood and may include psychotic symptoms or catatonia, leading to a substantial decline in functioning and quality of life (1–4). Although recent consensus criteria (see **Appendix 1**) have improved diagnostic consistency, the underlying etiopathogenesis remains poorly understood.

Before the publication of these criteria (2), knowledge about DSRD was largely derived from small case series and heterogeneous observational descriptions. Prior studies suggested that many individuals experience adverse life events or psychosocial stressors shortly before symptom onset (5–9), including developmental transitions, illness or death of a close figure, separation, medical events, residential changes, or exposure to stress or trauma. However, available evidence has been inconsistent, and systematic, multicenter data, particularly in adolescents and adults, are limited, hindering the ability to determine whether specific categories of stressors reliably precede DSRD.

Psychosocial stressors are known to influence psychiatric and medical conditions by increasing allostatic load and challenging adaptive capacity (1, 10). This framework supports the hypothesis that acute or chronic stress may be temporally associated with DSRD onset and could represent a relevant contextual factor; however, available evidence (including the present study) remains observational and does not support causal conclusions.

Although the syndrome shares phenomenological features with stress-related and reactive psychiatric presentations, its nosological boundaries remain debated, and underlying mechanisms are not yet fully elucidated (11–13). Given these gaps, there is a need for structured, systematic characterization of psychosocial stressors occurring in close temporal proximity to symptom onset in DSRD, particularly in clinical settings serving adolescents and adults with DS.

Therefore, the aim of the present study was to examine the prevalence, categories, and timing of psychosocial stressors occurring within ≤6 months before symptom onset in a consecutive cohort diagnosed with DSRD at two specialized referral clinics, and to describe the associated clinical presentation and functional severity at initial assessment. Consistent with the descriptive design, we frame these findings as hypothesis-generating, not establishing causality.

## Materials and methods

### Organizational setting of the study

This retrospective descriptive cohort study was conducted at two specialized referral centers in Madrid: The Department of Psychiatry at Clínica Universidad de Navarra, which includes a dedicated clinic for psychiatric care of individuals with intellectual disabilities, and the Adult DS Outpatient Clinic within the Internal Medicine Department at Hospital Universitario de La Princesa. Since 2005, the Adult DS Clinic has provided care for adolescents and adults with DS, with over 1,900 individuals assessed by 2024. The study period spanned from September 2021 to December 2024. Written informed consent was obtained from all participants or their legal guardians.

### Study population

We included all consecutive adolescents and adults diagnosed with DSRD during the study period. Diagnosis was established according to international expert consensus criteria (2; see **Appendix 1**). Exclusion criteria were: (a) age outside the 11–45 range; (b) regression attributable to autism spectrum disorder; (c) diagnosis of Alzheimer’s disease (given its increased prevalence in individuals with DS and potential symptom overlap); and (d) symptoms attributable to other organic or primary psychiatric conditions. Exclusion of primary psychiatric disorders was based on comprehensive clinical evaluation in all cases.

The upper age limit of 45 years was set to minimize diagnostic overlap with Alzheimer’s disease, whose prevalence increases markedly in individuals with Down syndrome from the fourth decade of life onward.

### Variables and determinations

Study variables were collected from the initial evaluation and, when available, follow-up visits. Variables included sociodemographic characteristics, clinical features, and functional severity, selected according to multidisciplinary expert recommendations (1, 2).

### Psychosocial stressor coding

Psychosocial stressors occurring within ≤6 months before symptom onset were identified from clinical records and caregiver interviews and coded using a standardized matrix (**Appendix 2**). Two independent reviewers, blinded to each other’s ratings and to clinical severity/outcome variables, coded stressors according to prespecified category definitions and decision rules; discrepancies were resolved by consensus. Missing or uncertain information was coded as “unknown” following predefined rules.

### Functional severity

Severity at the initial assessment was operationalized as global functional impairment relative to premorbid baseline. Severity was estimated as the percentage of preserved functioning, relative to the individual’s premorbid level (considered 100%), based on a structured clinician–caregiver review of changes in key functional domains (activities of daily living, communication, autonomy, social participation, and occupational/educational engagement). Anchors and operational criteria used for this consensus-based estimate are provided (see **Supplementary Table 1**).

Estimates were reached by consensus between the evaluating clinician and the primary caregiver. Given the absence of validated severity instruments specific to DSRD, this measure is reported descriptively and interpreted cautiously.

### Statistical analysis

**Figure 1:**
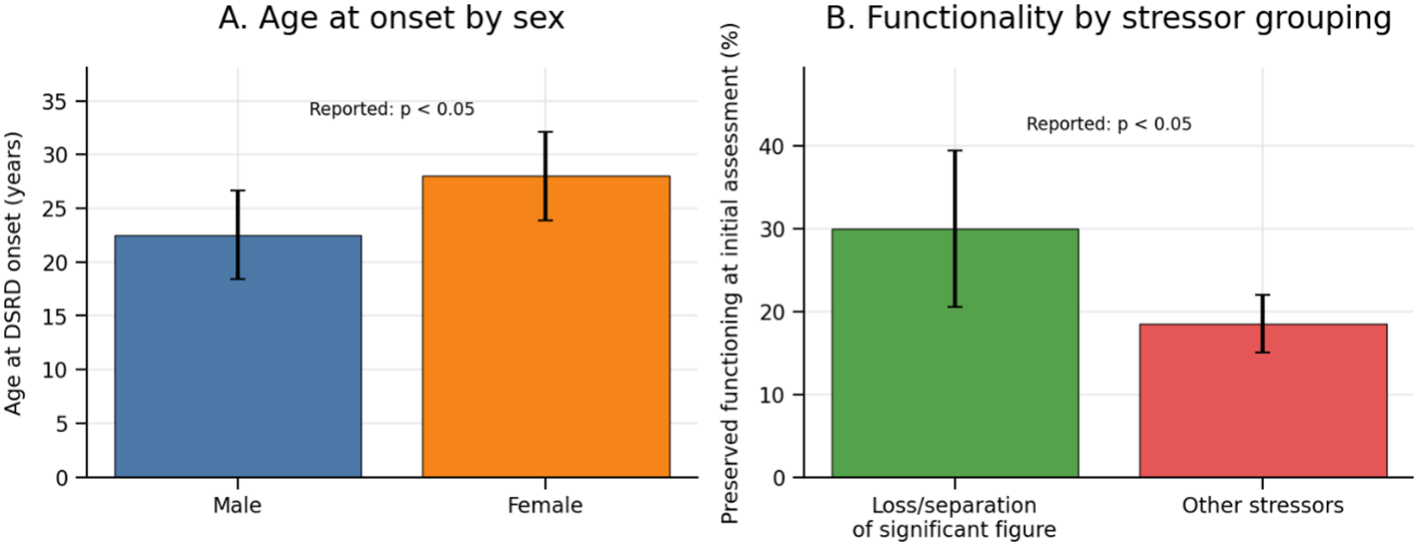
Graphical display of exploratory inferential comparisons. **(A)** Age at DSRD onset by sex (mean with 95% CI). **(B)** Preserved functioning at initial assessment (%) comparing participants whose psychosocial stressor involved loss/separation of a close family member or significant figure vs those with other stressor categories (mean with 95% CI).

The demographic and clinical features of the patients were summarized using means, standard deviations, frequencies, and percentages. The Shapiro–Wilk test was used to assess normality. Group comparisons for quantitative variables were conducted using either the Student’s t test or the Mann–Whitney test. For qualitative variables, the Pearson chi-square test or Fisher’s exact test was used. A p-value < 0.05 was considered statistically significant. All analyses were performed in Stata 18 (StataCorp LLC, College Station, TX). Confidence intervals for proportions were reported using the exact binomial (Clopper–Pearson) method; continuous variables were summarized as mean ± SD or median (IQR), as appropriate. This is a descriptive, hypothesis-generating study, and no *a priori* sample size calculation was performed. Exploratory inferential results are summarized in **Table 1** and **Figure 1**.

**Table 1:** Exploratory inferential analyses referenced in results sections.

Outcome	Groups compared	Test	Effect estimate	p-value	Notes
Age at DSRD onset (years)	Male (n=17) vs Female (n=17)	Student’s t test	22.5 ± 8.0 vs 28.0 ± 8.0	p < 0.05	Earlier onset in males.
Preserved functioning at initial assessment (%)	Loss/separation stressor group (n=18) vs other stressors (n=16)	Student’s t test	30.0% ± 19.0% vs 18.5% ± 6.6%	p < 0.05	Milder impairment in loss/separation group.

### Ethical considerations

The study adhered to the ethical principles outlined in the Declaration of Helsinki (14), followed Good Clinical Practice guidelines, and complied with local and national laws. Data confidentiality was maintained throughout, in line with LOPDGDD 3/2018. The project received approval from the IRB of Clínica Universidad de Navarra (2023.006) and the Hospital Universitario de La Princesa (registration number 5006).

## Results

### General and sociodemographic characteristics

Between September 2021 and December 2024, 34 individuals with DSRD were identified: 18 from the psychiatry clinic and 16 from the internal medicine department. Baseline demographic and clinical characteristics are summarized in **Table 2**. The mean age at DSRD onset was 25.5 ± 8 years (range: 11–43 years). Males had an earlier mean age at onset than females (mean age: 22.5 ± 8 years vs. 28 ± 8 years; p < 0.05; see **Table 1**, **Figure 1**). The cohort had an equal sex distribution (50% male, 50% female).

**Table 2:** Baseline demographic and clinical characteristics of the cohort (N = 34).

Characteristic	Value
Study period	September 2021 – December 2024
Referral sites (cases)	Psychiatry clinic: 18; Internal medicine clinic: 16
Age at DSRD onset (years), mean ± SD (range)	25.5 ± 8 (11–43)
Sex, n (%)	Male: 17 (50%); Female: 17 (50%)
Level of intellectual disability, n (%)	Mild: 8 (24%); Moderate: 20 (59%); Severe: 5 (15%); Unknown: 1 (3%)
Diagnostic delay: symptom onset → initial diagnostic consultation, median months (IQR)	36 (24–72)
Functional severity at initial assessment (estimated % of premorbid functioning), mean ± SD	25% ± 15%

Percentages are calculated using the number of participants with available data as the denominator. Individuals with regression attributable to autism spectrum disorder were excluded. Prior psychiatric history was not systematically ascertained in this retrospective cohort and therefore could not be reliably summarized.

Most participants (91%, n = 31) were born in Spain and lived with their parents and/or siblings in the family home. The most common level of intellectual disability was moderate (59%, n = 20), followed by mild (24%, n = 8). Among 30 individuals with available data, 60% (n = 18) attended special education and 40% (n = 12) attended regular schools. The most frequent daily activity was attendance at an Occupational Therapy Center (41%, n = 14), followed by a Day Center (26%, n = 9).

### Clinical and diagnostic characteristics

The median duration from symptom onset to initial diagnostic consultation was 36 months (interquartile range: 24–72 months). The most common symptom, present in all individuals, was withdrawal or social isolation. Other prevalent symptoms included psychomotor slowing (97%, n = 33), loss of previously acquired abilities or functional decline (94%, n = 32), lack of motivation or initiative (94%, n = 32), and decline in verbal communication (88%, n = 30). Additional symptoms reported by over half of the cohort included sadness or apathy (79%, n = 27), reduced attention span (68%, n = 23), difficulty in planning or organizing daily activities (62%, n = 21), catatonia as defined by DSM-5 (59%, n = 20), stereotypies or tics (59%, n = 20), insomnia (53%, n = 18), compulsive or ritual behaviors (50%, n = 17), and psychotic symptoms (50%, n = 17). A detailed symptomatic profile is presented in **Table 3**.

**Table 3:** Symptom distribution within the cohort of patients with DSRD at initial assessment.

Symptom	Number of subjects (N)	Percentage (%)	95% CI
Social withdrawal or avoidance of contact	34	100%	90-100
Psychomotor slowing	33	97%	85-100
Loss of abilities or functional deterioration	32	94%	80-99
Loss of initiative or motivation	32	94%	80-99
Decline in verbal communication	30	88%	73-97
Sadness or apathy	27	79%	62-91
Difficulty maintaining attention	23	68%	50-83
Difficulty with planning	21	62%	44-78
Catatonia according to DSM-5 criteria	20	59%	41-75
Stereotypies or tics	20	59%	41-75
Insomnia	18	53%	35-70
Compulsive or ritualized behaviors	17	50%	32-68
Psychotic symptoms: delusions and/or hallucinations	17	50%	32-68
Weight loss	16	47%	30-65
Loss of eye contact	14	41%	25-59
Fatigue	12	35%	20-54
Anorexia/Hyporexia	10	29%	15-48
Guilt ideas or negative thoughts	10	29%	15-48
Difficulty recalling memories	9	26%	13-44
Thoughts of death and/or somatic complaints	9	26%	13-44
Hyperactivity or disruptive behaviors	9	26%	13-44
Urinary incontinence	7	20%	9-38
Hyperphagia	5	15%	5-31
Weight gain	5	15%	5-31
Syncope or dizziness	3	9%	2-24
Hypersomnia	2	6%	1-20

95% CI calculated using the exact binomial (Clopper–Pearson) method; total N = 34.

At initial assessment, the mean estimated functionality observed in the cohort was 25% ± 15. This indicates marked functional impairment at the time of first specialized evaluation.

### Analysis of adverse psychosocial events

Of the 34 cases, 29 (85%) experienced at least one adverse life event from categories commonly identified in prior research before DSRD onset. Including additional stressful factors not previously categorized (e.g., the coronavirus pandemic or witnessing the terrorist attack), 31 individuals (91%, 95% CI 76–98) had at least one adverse psychosocial stressor within six months before symptom onset.

The most frequent adverse psychosocial experiences were the death or illness of a loved one (35%, 95% CI 20–54; n = 12), followed by separation or displacement of a close figure (32%, 95% CI 17–51; n = 11). In 53% of cases (n = 18), the identified stressor involved a close family member, loved one, or reference figure, most commonly a parent’s illness or loss of contact with a support figure. Less common factors included relocation (12%, 95% CI 3–28; n = 4) and awareness of personal disability or limitations (6%, 95% CI 1–20; n = 2). Multiple adverse life factors were present in 65% of subjects (95% CI 46–80; n = 22). Prevalence rates for these adverse psychosocial experiences are detailed in **Table 4**.

**Table 4:** Psychosocial stressors within ≤6 months before DSRD onset (N = 34).

Category	N	Percentage (%)	95% CI
Any psychosocial stressor (≥1, any category)	31	91	76-98
More than one stressor (≥2 categories/events)	22	65	46-80
Death or illness of a family member or close person	12	35	20-54
Separation/displacement of a significant figure	11	32	17–51
Transition to adolescence/adulthood or life cycle changes	6	18	7-35
Overstimulation, excessive environmental demands	5	15	5–31
Medical illness or surgery	5	15	5–31
Abuse, bullying or aggression	5	15	5–31
Change of residence	4	12	3-28
Awareness of intellectual disability	2	6	1-20
Other	2	6	1-20

Stressors were coded from clinical records/caregiver interviews using the prespecified coding matrix (**Appendix 2**). Categories are not mutually exclusive; participants could have more than one psychosocial stressor. 95% CI calculated using the exact binomial (Clopper–Pearson) method.

### Relationship between severity and psychosocial stressors

We explored the relationship between clinical severity and specific psychosocial stressors. Changes in residence (mean functionality: 12% ± 10), awareness of one’s own disability (17.5% ± 3.5), and experiences of abuse or aggression (21% ± 7) were observed alongside lower estimated functioning (greater severity) than other stressor categories. However, the small sample size limited a detailed investigation of this potential connection. Conversely, participants whose stressor involved loss or separation from a close family member or significant figure (53%, n = 18) showed milder impairment at initial assessment compared to those with other adverse psychosocial factors (mean functionality: 30% ± 19 vs. 18.5% ± 6.6; p < 0.05; see **Table 1**, **Figure 1**). These findings should be interpreted with caution due to the descriptive nature of the study and limited sample size.

## Discussion

This study presents a descriptive analysis of 34 individuals diagnosed with DSRD at two specialized clinics. Our findings indicate a high prevalence of adverse psychosocial events in the six months preceding symptom onset, with bereavement, illness, or separation from a loved one, being the most frequently reported stressors. These results are consistent with previous studies reporting frequent psychosocial stressors in temporal proximity to DSRD onset (1, 5, 6, 9).

The mean age at onset in our cohort (25.5 years) is higher than that reported in earlier series, reflecting the adult focus of our referral centers. While some evidence suggests earlier onset in males, previous studies have not consistently identified a sex-related pattern (1, 5). Our results underscore that DSRD can occur at older ages than initially described, emphasizing the need for ongoing diagnostic vigilance throughout adulthood.

A notable finding is the substantial delay between symptom onset and initial diagnostic consultation, with a median of 36 months. This diagnostic delay has also been reported in other cohorts (1, 5, 7, 9) and may contribute to increased functional decline and reduced quality of life. Raising clinical awareness and refining assessment pathways may help shorten diagnostic delays and support timely clinical evaluation, although our data do not allow conclusions about the impact of earlier recognition on outcomes.

The clinical profile observed in our cohort aligns with existing literature, confirming a constellation of symptoms including social withdrawal, psychomotor slowing, loss of previously acquired abilities, diminished motivation, and decline in verbal communication. Additional symptoms such as sadness, apathy, attention deficits, catatonia, stereotypies, insomnia, compulsive behaviors, and psychotic features were also prevalent. Variability in symptom presentation may reflect differences in clinical assessment, patient age, and the multidisciplinary nature of our sample.

Our analysis suggests that psychosocial context may be relevant to consider when individuals with DS present with acute or subacute regression, particularly following bereavement, illness, or separation from close figures. While this finding may serve as a basis for recommending clinical vigilance, it should not be interpreted as evidence justifying the adoption of preventive strategies, given the descriptive nature of the study and the absence of a comparison group. Although some previous studies have suggested that certain stressors may be associated with better recovery following treatment (7, 9), our sample size limits definitive conclusions regarding the relationship between specific stressors and clinical outcomes.

Comparisons with other cohorts, such as those described by Wang et al. (5) and Miles et al. (9), reveal both similarities and differences in the types of psychosocial stressors reported. Wang et al. emphasized transitions to adulthood and school, while Miles highlighted traumatic events and acute medical illnesses. Our findings suggest that bereavement and medical illness in loved ones are commonly reported in temporal proximity to DSRD onset in this cohort. Any interpretation regarding prognosis or clinical improvement should be made cautiously and require dedicated outcome focused studies.

The high frequency of these factors may place DSRD within the spectrum of reactive psychiatric illnesses, but the exact prevalence and etiopathogenesis remain unknown. The interplay between acute or chronic stressors, individual biology, and broader psychosocial context likely influences both the onset and course of DSRD.

These findings also have implications for clinicians who care for individuals with DS. The marked diagnostic delays observed in our cohort highlight the need to raise awareness of DSRD among clinicians, to improve access to specialized assessment, and to integrate psychosocial screening into routine care pathways for adolescents and adults with DS. Systematic consideration of psychosocial context may assist clinicians in the comprehensive assessment of suspected DSRD; however, the extent to which these influences outcomes cannot be determined from the present data.

## Strengths and limitations

This study benefits from one of the largest DSRD cohorts published to date and includes a comprehensive assessment of both clinical features and psychosocial variables. Data were obtained from two referral centers with longstanding expertise in the care of individuals with DS, which enhances the robustness and clinical relevance of the findings. Detailed symptom characterization also expands the current phenotypic description of DSRD and may contribute to improved diagnostic recognition.

Several limitations should be acknowledged. Adverse life events are common in both the general population and individuals with Down syndrome; in the absence of a control group drawn from the same source population, we cannot determine whether the observed stressors are specific to DSRD. Accordingly, these findings should be interpreted as descriptive and temporally associated, rather than indicative of specificity or causality. Prior psychiatric history was not systematically ascertained in this retrospective cohort and therefore could not be reliably summarized. Furthermore, diagnostic delay and the use of a non-validated, consensus-based estimate of preserved functioning may limit comparability across studies and introduce variability; however, no validated DSRD-specific severity instruments are currently available, and this pragmatic approach was used to capture global functional impairment in routine clinical practice. Future studies should incorporate validated standardized scales for activities of daily living and psychiatric symptomatology to allow more reliable severity assessment and improve cross-study comparability. Finally, the relatively older age of the cohort may have influenced the distribution of certain psychosocial stressors, particularly those related to illness or loss among aging parents or other reference figures.

## Conclusions

This study expands current knowledge of DSRD by characterizing a cohort of 34 individuals evaluated at two specialized clinics. We found a high prevalence of adverse psychosocial experiences—particularly bereavement, illness, or separation from close figures in the six months preceding symptom onset. These findings suggest that adverse psychosocial experiences are frequently present in temporal proximity to symptom onset in DSRD. In clinical practice, awareness of recent major stressors may be useful as contextual information when evaluating individuals with DS presenting with regression, while acknowledging that these observations do not establish specificity or causality.

The detailed symptom profile described here may support clinical recognition and may help refine descriptive phenotyping of DSRD. Although this study does not allow causal conclusions or statements about prevention, it reinforces the importance of incorporating psychosocial context into clinical assessment. Future research using controlled designs and validated outcome measures is needed to clarify specificity, mechanisms, and clinically actionable risk stratification.

## Data Availability

The raw data supporting the conclusions of this article will be made available by the authors, without undue reservation.

## Supplementary material

Supplementary Table 1 Criteria for consensus-based preserved functioning estimate at initial assessment.

## Appendix

**Table:** Appendix 1

Consensus recommendations for diagnosing Down Syndrome Regression Disorder			
Category	Criteria	Possible DSRD	DSRD
Symptom onset	Onset of neurologic, psychiatric or mixed symptoms over a period of <12 weeks in a previously healthy individual with DS	Yes	Yes (3 symptom clusters present)
Clinical evidence of neurologic dysfunction	Altered mental status or behavioral dysregulation: Anorexia, decreased oral intake or hyperphagia. Confusion, disorientation. Inappropriate laughter. Cognitive decline: Apathy. Abulia and/or avolition. Developmental regression with or without new autistic features: Social withdrawal. Loss of previously acquired developmental milestones. Inability to perform activities of daily living. Stereotypy. Rigidity around routine changes. Decreased eye contact. Insomnia or circadian rhythm disruption: Language deficits: Expressive and/or receptive aphasia: Global aphasia (mutism). Whispered speech. Movement disorder (excluding tics): Catatonia. Bradykinesia. Freezing. Gait disturbance.		
Exclusion of other etiologies	Reasonable exclusion of alternative causes of DSRD, including other systemic and central nervous system disorders. Other primary psychiatric disorders are also considered exclusionary.		

## Appendix 2

**Table:** Coding matrix and rules for psychosocial stressors.

Category	Definition
Bereavement/Illness of Close Person	Serious illness or death of a family member or close figure.
Separation/Displacement	Prolonged absence or loss of contact with a caregiver or significant person.
Transition to Adolescence/Adulthood or Life Cycle Changes	Major transitions such as finishing school, starting work, or moving to adult services.
Overstimulation/Excessive Demands	Sudden increase in environmental demands, noise, or expectations.
Medical Illness/Surgery	Acute medical events, hospitalizations, or surgical interventions.
Abuse/Bullying/Aggression	Exposure to bullying, aggression, or abuse.
Change of Residence	Moving home or changing care setting.
Awareness of Disability	New or persistent preoccupation with one’s own limitations.
Other	Any significant event not captured above (describe in free text).
Rules	
Only events occurring within ≤6 months before the first DSRD symptoms were coded	
Multiple events per case were allowed and coded independently	
Coding was based on medical records and caregiver interviews	
Uncertainty or missing data was coded as “unknown”	
All coders followed a prespecified manual	
