# Supplementary material for: Psychosocial stressors prior to Down Syndrome Regression Disorder: findings from two referral clinics
Source: Front Psychiatry. 2026 Jul 7;17:1799799. doi: 10.3389/fpsyt.2026.1799799 (PMC13386263; doi:10.3389/fpsyt.2026.1799799)
Supplement: Supplementary Table 1 — Criteria for consensus-based preserved functioning estimate at initial assessment. [file DataSheet1.docx]

**SUPPLEMENTARY MATERIAL**

**Supplementary Table S1**. Criteria for consensus-based preserved functioning estimate at initial assessment.

| **Preserved functioning (%)** | **Qualitative anchor** | **Operational descriptors (examples)** |
| --- | --- | --- |
| 100% | Normal (baseline; not ill) | Functioning comparable to premorbid baseline across domains; no clinically meaningful decline |
| 80% | Mildly ill | Mild changes in 1–2 domains (e.g., initiative/efficiency or social participation); independent in basic ADLs with functional communication; minor prompting/support only |
| 60% | Moderately ill | Clear decline in multiple domains; needs regular prompting/supervision for some instrumental ADLs (planning/organization); reduced autonomy/social participation; communication reduced but functional; occupational/educational engagement reduced |
| 40% | Markedly ill | Marked global impairment; frequent assistance for basic ADLs and/or consistent supervision; prominent withdrawal and reduced communication; unable to sustain usual occupational/educational activities |
| 20% | Severely ill | Severe dependence across domains; extensive assistance for basic ADLs; communication markedly reduced/intermittently absent; minimal autonomy and very limited social participation; severe motor/behavioral symptoms may be present (e.g., catatonia). |
| <10% | Among the most extremely ill patients | Near-complete loss of premorbid functioning; profound dependence for ADLs; minimal/absent functional communication; requires continuous care/supervision |

Note: ADL = activities of daily living.
